# Supplementary material for: The impact of varying the number and selection of conditions on estimated multimorbidity prevalence: A cross-sectional study using a large, primary care population dataset
Source: PLoS Med. 2023 Apr 4;20(4):e1004208. doi: 10.1371/journal.pmed.1004208 (PMC10072475; doi:10.1371/journal.pmed.1004208)
Supplement: S1 Table — (DOCX) [file pmed.1004208.s001.docx]

# Supplementary Table 1. List of chronic conditions examined, and codes used to define those morbidities

| **Condition used in this analysis** | **Condition subsets (to map to code lists)** | **Codes used or code list source** |
| --- | --- | --- |
| Addison’s Disease |  | Read v2 codes: A176. C154. C1540 C1541 C1546 C154z  ICD10 codes: E27.1 E27.2 |
| Alcohol misuse |  | https://phenotypes.healthdatagateway.org/phenotypes/PH94/version/188/detail/#home |
| Ankylosing spondylitis |  | https://phenotypes.healthdatagateway.org/phenotypes/PH99/version/198/detail/#home |
| Anxiety |  | https://phenotypes.healthdatagateway.org/phenotypes/PH104/version/208/detail/#home |
| Aplastic anaemia |  | https://phenotypes.healthdatagateway.org/phenotypes/PH105/version/210/detail/#home |
| Asthma |  | https://phenotypes.healthdatagateway.org/phenotypes/PH109/version/218/detail/#home |
| Atrial fibrillation |  | https://phenotypes.healthdatagateway.org/phenotypes/PH36/version/72/detail/#home |
| Autism |  | https://phenotypes.healthdatagateway.org/phenotypes/PH110/version/220/detail/#home |
| Benign prostatic hyperplasia |  | https://phenotypes.healthdatagateway.org/phenotypes/PH39/version/78/detail/#home |
| Bipolar affective disorder |  | https://phenotypes.healthdatagateway.org/phenotypes/PH38/version/76/detail/#home |
| Bronchiectasis |  | https://phenotypes.healthdatagateway.org/phenotypes/PH128/version/256/detail/#home |
| Cardiomyopathy | Dilated cardiomyopathy  Hypertrophic cardiomyopathy  Cardiomyopathy – other | https://phenotypes.healthdatagateway.org/phenotypes/PH145/version/290/detail/#home  https://phenotypes.healthdatagateway.org/phenotypes/PH185/version/370/detail/#home  https://phenotypes.healthdatagateway.org/phenotypes/PH129/version/258/detail/#home |
| Cerebral palsy |  | https://phenotypes.healthdatagateway.org/phenotypes/PH132/version/264/detail/#home |
| Chronic kidney disease | Chronic kidney disease (laboratory result defined)  End stage renal disease | https://phenotypes.healthdatagateway.org/phenotypes/PH42/version/1527/detail/#home  https://phenotypes.healthdatagateway.org/phenotypes/PH45/version/90/detail/#home |
| Chronic obstructive pulmonary disease |  | https://phenotypes.healthdatagateway.org/phenotypes/PH43/version/86/detail/#home |
| Coeliac disease |  | https://phenotypes.healthdatagateway.org/phenotypes/PH141/version/282/detail/#home |
| Coronary heart disease (CHD) | Myocardial infarction  Stable angina  Unstable angina  CHD not otherwise specified | https://phenotypes.healthdatagateway.org/phenotypes/PH215/version/430/detail/#home  https://phenotypes.healthdatagateway.org/phenotypes/PH315/version/630/detail/#home  https://phenotypes.healthdatagateway.org/phenotypes/PH329/version/658/detail/#home  https://phenotypes.healthdatagateway.org/phenotypes/PH41/version/82/detail/#home |
| Cystic fibrosis |  | https://phenotypes.healthdatagateway.org/phenotypes/PH40/version/80/detail/#home |
| Dementia |  | https://phenotypes.healthdatagateway.org/phenotypes/PH148/version/296/detail/#home |
| Depression |  | https://phenotypes.healthdatagateway.org/phenotypes/PH149/version/298/detail/#home |
| Diabetes mellitus other or not specified |  | https://phenotypes.healthdatagateway.org/phenotypes/PH152/version/304/detail/ |
| Diverticular disease |  | https://phenotypes.healthdatagateway.org/phenotypes/PH154/version/308/detail/#home |
| Eating disorders |  | https://phenotypes.healthdatagateway.org/phenotypes/PH159/version/318/detail/#home |
| Endometriosis |  | https://phenotypes.healthdatagateway.org/phenotypes/PH162/version/324/detail/ |
| Epilepsy |  | https://phenotypes.healthdatagateway.org/phenotypes/PH165/version/330/detail/#home |
| Erectile dysfunction |  | https://phenotypes.healthdatagateway.org/phenotypes/PH44/version/88/detail/#home |
| Giant cell arteritis |  | https://phenotypes.healthdatagateway.org/phenotypes/PH46/version/92/detail/#home |
| Gout |  | https://phenotypes.healthdatagateway.org/phenotypes/PH177/version/354/detail/#home |
| Haematological malignancy |  | https://phenotypes.healthdatagateway.org/phenotypes/PH60/version/120/detail/#home  https://phenotypes.healthdatagateway.org/phenotypes/PH63/version/126/detail/#home  https://phenotypes.healthdatagateway.org/phenotypes/PH68/version/136/detail/#home  https://phenotypes.healthdatagateway.org/phenotypes/PH186/version/372/detail/  https://phenotypes.healthdatagateway.org/phenotypes/PH199/version/398/detail/  https://phenotypes.healthdatagateway.org/phenotypes/PH239/version/478/detail/#home |
| Hearing loss |  | Read version 2 codes: 1C13300 2BL3.00 2BL4.00 F580100 F591400 F591600 F591C00 F592100 F598.00 1C17.00 2DG..00 2DH0.00 7311A00 7317C00 7320 7319600 7319 8D23.00 8D26.00 8D2..12 8M41.00 Z8B5300 Z8B5500 Z911100 Z911.00 8D2..12 ZV45G00 ZV45N00 ZV53200 ZV53D00 2BL5.00 F591900 F591A00  ICD-10 codes: H90, Z45.3, Z46.1, Z97.4 |
| Heart block and bundle branch block | Atrioventricular block second degree  Bifascicular block  Trifascicular block  Atrioventricular block third degree  Left bundle branch block  Right bundle branch block | https://phenotypes.healthdatagateway.org/phenotypes/PH114/version/228/detail/  https://phenotypes.healthdatagateway.org/phenotypes/PH125/version/250/detail/  https://phenotypes.healthdatagateway.org/phenotypes/PH324/version/648/detail/#home  https://phenotypes.healthdatagateway.org/phenotypes/PH115/version/230/detail/  https://phenotypes.healthdatagateway.org/phenotypes/PH57/version/114/detail/#home  https://phenotypes.healthdatagateway.org/phenotypes/PH78/version/156/detail/#home |
| Heart failure |  | https://phenotypes.healthdatagateway.org/phenotypes/PH182/version/364/detail/#home |
| Heart valve disease | Rheumatic valve disorder  Non-rheumatic aortic valve disorder  Non-rheumatic mitral valve disorder  Multiple valve disorder | https://phenotypes.healthdatagateway.org/phenotypes/PH81/version/162/detail/  https://phenotypes.healthdatagateway.org/phenotypes/PH219/version/438/detail/#home  https://phenotypes.healthdatagateway.org/phenotypes/PH220/version/440/detail/#home  https://phenotypes.healthdatagateway.org/phenotypes/PH212/version/424/detail/#home |
| Human immunodeficiency virus |  | https://phenotypes.healthdatagateway.org/phenotypes/PH184/version/368/detail/#home |
| Hyperparathyroidism |  | https://phenotypes.healthdatagateway.org/phenotypes/PH76/version/152/detail/ |
| Hypertension |  | https://phenotypes.healthdatagateway.org/phenotypes/PH189/version/378/detail/#home |
| Inflammatory arthritis | Juvenile Arthritis  Psoriatic Arthritis  Reactive Arthritis  Rheumatoid Arthritis | https://phenotypes.healthdatagateway.org/phenotypes/PH196/version/392/detail/  https://phenotypes.healthdatagateway.org/phenotypes/PH80/version/160/detail/ |
| Inflammatory bowel disease | Crohn’s Disease  Ulcerative colitis | https://phenotypes.healthdatagateway.org/phenotypes/PH144/version/288/detail/  https://phenotypes.healthdatagateway.org/phenotypes/PH326/version/652/detail/#home |
| Intellectual disability | Down Syndrome  Other intellectual disability | https://phenotypes.healthdatagateway.org/phenotypes/PH156/version/312/detail/  https://phenotypes.healthdatagateway.org/phenotypes/PH192/version/384/detail/ |
| Liver disease | Alcoholic liver disease  Autoimmune liver disease  Chronic viral hepatitis  Cirrhosis and liver fibrosis  Fatty liver  Liver failure  Oesophageal varices  Portal hypertension | https://phenotypes.healthdatagateway.org/phenotypes/PH202/version/404/detail/  https://phenotypes.healthdatagateway.org/phenotypes/PH111/version/222/detail/  https://phenotypes.healthdatagateway.org/phenotypes/PH137/version/274/detail/  https://phenotypes.healthdatagateway.org/phenotypes/PH140/version/280/detail/  https://phenotypes.healthdatagateway.org/phenotypes/PH168/version/336/detail/  https://phenotypes.healthdatagateway.org/phenotypes/PH203/version/406/detail/  https://phenotypes.healthdatagateway.org/phenotypes/PH334/version/668/detail/#home  https://phenotypes.healthdatagateway.org/phenotypes/PH243/version/486/detail/#home |
| Lupus Erythematosus |  | https://phenotypes.healthdatagateway.org/phenotypes/PH83/version/166/detail/#home |
| Motor Neurone Disease |  | https://phenotypes.healthdatagateway.org/phenotypes/PH62/version/124/detail/#home |
| Multiple sclerosis |  | https://phenotypes.healthdatagateway.org/phenotypes/PH63/version/126/detail/#home |
| Myasthenia gravis |  | https://phenotypes.healthdatagateway.org/phenotypes/PH213/version/426/detail/#home |
| Neuropathic bladder |  | https://phenotypes.healthdatagateway.org/phenotypes/PH218/version/436/detail/#home |
| Obsessive compulsive disorder |  | https://phenotypes.healthdatagateway.org/phenotypes/PH223/version/446/detail/#home |
| Obstructive and reflux uropathy |  | https://phenotypes.healthdatagateway.org/phenotypes/PH222/version/444/detail/ |
| Osteoarthritis |  | https://phenotypes.healthdatagateway.org/phenotypes/PH63/version/126/detail/#home |
| Osteoporosis |  | https://phenotypes.healthdatagateway.org/phenotypes/PH225/version/450/detail/#home |
| Paralysis |  | Read version 2 codes: 2835 F241100 F141.00 F241.00 F241000 F230000 F240.11 F240100 F240000 F232.11 F232.00 2836 F240.00 F242.00 F230.00 F230z00 2837 F230100  ICD-10 codes: G82, G83.0, G80.8, G80.1, G80.3 |
| Parkinson’s Disease |  | https://phenotypes.healthdatagateway.org/phenotypes/PH77/version/154/detail/ |
| Paroxysmal tachycardias | Supraventricular tachycardia  Ventricular tachycardia | https://phenotypes.healthdatagateway.org/phenotypes/PH84/version/168/detail/#home  https://phenotypes.healthdatagateway.org/phenotypes/PH90/version/180/detail/#home |
| Peripheral artery disease |  | https://phenotypes.healthdatagateway.org/phenotypes/PH235/version/470/detail/#home |
| Peripheral neuropathy | Peripheral neuropathy  Autonomic neuropathy | https://phenotypes.healthdatagateway.org/phenotypes/PH235/version/470/detail/#home  https://phenotypes.healthdatagateway.org/phenotypes/PH112/version/224/detail/  https://phenotypes.healthdatagateway.org/phenotypes/PH155/version/310/detail/#home (selected codes) |
| Polymyalgia rheumatica |  | https://phenotypes.healthdatagateway.org/phenotypes/PH74/version/148/detail/#home |
| Primary malignancy | Individual cancers site/type | https://phenotypes.healthdatagateway.org/phenotypes/PH250/version/500/detail/#home  https://phenotypes.healthdatagateway.org/phenotypes/PH252/version/504/detail/#home  https://phenotypes.healthdatagateway.org/phenotypes/PH256/version/512/detail/#home  https://phenotypes.healthdatagateway.org/phenotypes/PH265/version/530/detail/#home  https://phenotypes.healthdatagateway.org/phenotypes/PH247/version/494/detail/#home  https://phenotypes.healthdatagateway.org/phenotypes/PH248/version/496/detail/#home  https://phenotypes.healthdatagateway.org/phenotypes/PH249/version/498/detail/#home  https://phenotypes.healthdatagateway.org/phenotypes/PH251/version/502/detail/#home  https://phenotypes.healthdatagateway.org/phenotypes/PH253/version/506/detail/#home  https://phenotypes.healthdatagateway.org/phenotypes/PH254/version/508/detail/#home  https://phenotypes.healthdatagateway.org/phenotypes/PH255/version/510/detail/#home  https://phenotypes.healthdatagateway.org/phenotypes/PH257/version/514/detail/#home  https://phenotypes.healthdatagateway.org/phenotypes/PH258/version/516/detail/#home  https://phenotypes.healthdatagateway.org/phenotypes/PH259/version/518/detail/#home  https://phenotypes.healthdatagateway.org/phenotypes/PH260/version/520/detail/#home  https://phenotypes.healthdatagateway.org/phenotypes/PH260/version/520/detail/#home  https://phenotypes.healthdatagateway.org/phenotypes/PH262/version/524/detail/  https://phenotypes.healthdatagateway.org/phenotypes/PH263/version/526/detail/#home  https://phenotypes.healthdatagateway.org/phenotypes/PH264/version/528/detail/#home  https://phenotypes.healthdatagateway.org/phenotypes/PH266/version/532/detail/#home  https://phenotypes.healthdatagateway.org/phenotypes/PH267/version/534/detail/#home  https://phenotypes.healthdatagateway.org/phenotypes/PH268/version/536/detail/#home  https://phenotypes.healthdatagateway.org/phenotypes/PH270/version/540/detail/#home  https://phenotypes.healthdatagateway.org/phenotypes/PH271/version/542/detail/#home  https://phenotypes.healthdatagateway.org/phenotypes/PH271/version/542/detail/#home |
| Primary thrombocytopenia |  | https://phenotypes.healthdatagateway.org/phenotypes/PH269/version/538/detail/#home |
| Psoriasis |  | https://phenotypes.healthdatagateway.org/phenotypes/PH273/version/546/detail/#home |
| Pulmonary fibrosis |  | https://phenotypes.healthdatagateway.org/phenotypes/PH276/version/552/detail/#home |
| Raynaud’s disease |  | https://phenotypes.healthdatagateway.org/phenotypes/PH277/version/554/detail/#home |
| Schizophrenia |  | https://phenotypes.healthdatagateway.org/phenotypes/PH285/version/570/detail/#home |
| Scleroderma |  | https://phenotypes.healthdatagateway.org/phenotypes/PH318/version/636/detail/#home |
| Secondary malignancy | Individual cancers site/type | https://phenotypes.healthdatagateway.org/phenotypes/PH292/version/584/detail/#home  https://phenotypes.healthdatagateway.org/phenotypes/PH290/version/580/detail/#home  https://phenotypes.healthdatagateway.org/phenotypes/PH291/version/582/detail/#home  https://phenotypes.healthdatagateway.org/phenotypes/PH294/version/588/detail/#home  https://phenotypes.healthdatagateway.org/phenotypes/PH293/version/586/detail/#home  https://phenotypes.healthdatagateway.org/phenotypes/PH295/version/590/detail/#home  https://phenotypes.healthdatagateway.org/phenotypes/PH289/version/578/detail/#home  https://phenotypes.healthdatagateway.org/phenotypes/PH297/version/594/detail/#home  https://phenotypes.healthdatagateway.org/phenotypes/PH299/version/598/detail/#home  https://phenotypes.healthdatagateway.org/phenotypes/PH298/version/596/detail/#home |
| Sick sinus syndrome |  | https://phenotypes.healthdatagateway.org/phenotypes/PH303/version/606/detail/#home |
| Sickle cell disease |  | https://phenotypes.healthdatagateway.org/phenotypes/PH304/version/608/detail/#home |
| Sjogren Syndrome |  | https://phenotypes.healthdatagateway.org/phenotypes/PH307/version/614/detail/#home |
| Sleep apnoea |  | https://phenotypes.healthdatagateway.org/phenotypes/PH309/version/618/detail/#home |
| Stroke and transient ischaemic attack | Ischaemic stroke  Transient ischaemic attack  Stroke not otherwise specified | https://phenotypes.healthdatagateway.org/phenotypes/PH56/version/112/detail/#home  https://phenotypes.healthdatagateway.org/phenotypes/PH88/version/176/detail/  https://phenotypes.healthdatagateway.org/phenotypes/PH84/version/168/detail/ |
| Substance misuse |  | https://phenotypes.healthdatagateway.org/phenotypes/PH317/version/634/detail/ |
| Thalassaemia |  | https://phenotypes.healthdatagateway.org/phenotypes/PH320/version/640/detail/#home |
| Thyroid disease | Hyperthyroidism  Hypothyroidism | https://phenotypes.healthdatagateway.org/phenotypes/PH322/version/644/detail/#home |
| Tubulo-interstitial nephropathy |  | https://phenotypes.healthdatagateway.org/phenotypes/PH89/version/178/detail/#home |
| Type 1 diabetes mellitus |  | https://phenotypes.healthdatagateway.org/phenotypes/PH152/version/304/detail/ (includes algorithm to define type) |
| Type 2 diabetes mellitus |  | https://phenotypes.healthdatagateway.org/phenotypes/PH152/version/304/detail/ (includes algorithm to define type) |
| Upper GI acid conditions | Barrett’s oesophagus  Gastro-oesophageal reflux disease  Oesophagitis and oesophageal ulcer  Gastritis and duodenitis  Peptic ulcer | https://phenotypes.healthdatagateway.org/phenotypes/PH118/version/236/detail/  https://phenotypes.healthdatagateway.org/phenotypes/PH48/version/96/detail/#home  https://phenotypes.healthdatagateway.org/phenotypes/PH224/version/448/detail/#home  https://phenotypes.healthdatagateway.org/phenotypes/PH175/version/350/detail/  https://phenotypes.healthdatagateway.org/phenotypes/PH326/version/652/detail/#home |
| Urinary Incontinence |  | https://phenotypes.healthdatagateway.org/phenotypes/PH330/version/660/detail/ |
| Urolithiasis |  | https://phenotypes.healthdatagateway.org/phenotypes/PH331/version/662/detail/ |
| Venous thromboembolic disease |  | https://phenotypes.healthdatagateway.org/phenotypes/PH338/version/676/detail/ |
| Visual impairment and blindness |  | Read version 2 codes: 1B75. 2B6A. 2B6B. 2B6S. 2B7A. 2B7B. 2B7S. 2BBo. 2BBr. 6688 6689 668D. 8F6.. 8F61. 8F62. 8HlE. 9m08. 9NfB. 9NlD. F4041 F4042 F49.. F490. F4900 F4901 F4902 F4904 F4906 F4909 F490z F491. F4910 F4911 F4913 F4914 F4915 F4917 F491z F492. F4920 F4922 F4923 F4924 F4925 F492z F493. F494. F495. F4950 F4951 F4952 F4953 F4954 F4955 F4956 F4958 F495A F495z F496. F4960 F4961 F4962 F4963 F4964 F4965 F4966 F496z F497. F498. F499. F49A. F49B. F49C. F49y. F49z. F49z0 F4H73  Fy1.. FyuL. SJ0z. Z96.. Z961. Z962. ZK74. ZN568 ZN56A ZRhO. ZRr6. ZV410  ICD-10 code: H54 |
