# Supplementary material for: The impact of varying the number and selection of conditions on estimated multimorbidity prevalence: A cross-sectional study using a large, primary care population dataset
Source: PLoS Med. 2023 Apr 4;20(4):e1004208. doi: 10.1371/journal.pmed.1004208 (PMC10072475; doi:10.1371/journal.pmed.1004208)
Supplement: S2 Table — (DOCX) [file pmed.1004208.s002.docx]

# Supplementary Table 2. Conditions in each condition-list as implemented in this study, and as stated in published condition-lists.

| **Condition-list** | **Condition implemented in this study (matched to published condition-lists)** | **Conditions as stated in published condition-lists** |
| --- | --- | --- |
| **Barnett (2012)[1]** | **41 conditions**  Hypertension | Hypertension |
|  | .. | Painful condition |
|  | Asthma | Asthma |
|  | Coronary heart disease | Coronary heart disease |
|  | Upper gastro-intestinal acid disorders | Treated dyspepsia |
|  | Diabetes – other  Type 1 diabetes mellitus  Type 2 diabetes mellitus | Diabetes |
|  | Thyroid disease | Thyroid disorders |
|  | Inflammatory arthritis  Lupus erythematosus  Sjogren syndrome  Scleroderma | Rheumatoid arthritis, other inflammatory polyarthropathies & systematic connective tissue disorders, |
|  | Hearing impairment | Hearing loss |
|  | Chronic obstructive pulmonary disease | Chronic obstructive pulmonary disease |
|  | .. | Irritable bowel syndrome |
|  | Primary malignancy  Haematological malignancy  Secondary malignancy | New diagnosis of cancer in last five years |
|  | .. | Treated constipation |
|  | Stroke and transient ischaemic attack | Stroke and transient ischaemic attack |
|  | Chronic kidney disease | Chronic kidney disease |
|  | Diverticulosis | Diverticular disease of intestine |
|  | Atrial fibrillation | Atrial fibrillation |
|  | Peripheral arterial disease | Peripheral vascular disease |
|  | Heart failure | Heart failure |
|  | Benign prostatic hyperplasia | Prostate disorders |
|  | .. | Glaucoma |
|  | Epilepsy | Epilepsy |
|  | Psoriasis | Psoriasis or eczema |
|  | Inflammatory bowel disease | Inflammatory bowel disease |
|  | .. | Migraine |
|  | Visual impairment | Blindness & low vision |
|  | .. | Chronic sinusitis |
|  | Bronchiectasis | Bronchiectasis |
|  | Parkinson’s disease | Parkinson’s disease |
|  | Multiple sclerosis | Multiple sclerosis |
|  | Liver disease | Viral hepatitis  Chronic liver disease |
|  | Dementia | Dementia |
|  | Schizophrenia  Bipolar affective disorder | Schizophrenia or bipolar disorder |
|  | Intellectual disability | Learning disability |
|  | Eating disorders | Anorexia or bulimia |
|  | Depression | Depression |
|  | Anxiety | Anxiety & other neurotic stress related & somatoform disorders |
|  | Alcohol misuse | Alcohol problems |
|  | Substance misuse | Other psychoactive substance misuse |
| **Charlson (1987)[2]** | **21 conditions**  Coronary heart disease | Myocardial infarction |
|  | Heart failure | Congestive heart failure |
|  | Peripheral arterial disease | Peripheral vascular disease |
|  | Stroke and transient ischaemic attack | Cerebrovascular disease |
|  | Paralysis | Hemiplegia or paraplegia |
|  | Chronic obstructive pulmonary disease | Chronic pulmonary disease |
|  | Inflammatory arthritis  Lupus erythematosus  Sjogren syndrome  Scleroderma | Rheumatologic disease |
|  | Upper GASTRO-INTESTINAL acid disorders | Peptic ulcer disease |
|  | Diabetes – other  Type 1 diabetes mellitus  Type 2 diabetes mellitus | Diabetes without chronic complications  Diabetes with chronic complications |
|  | Chronic kidney disease | Moderate or severe renal disease |
|  | Primary malignancy  Haematological malignancy | Any malignancy, including leukaemia and lymphoma |
|  | Secondary malignancy | Metastatic solid tumour |
|  | Liver disease | Mild liver disease  Moderate to severe liver disease |
|  | HIV | AIDS/HIV |
|  | Dementia | Dementia |
| **Diederichs (2011)[3]** | **18 conditions**  Primary malignancy  Haematological malignancy  Secondary malignancy | Cancer |
|  | Diabetes – other  Type 1 diabetes mellitus  Type 2 diabetes mellitus | Diabetes mellitus |
|  | Hypertension | Hypertension |
|  | Coronary heart disease | Myocardial infarction  Chronic ischemic heart disease |
|  | Atrial fibrillation  Paroxysmal tachycardias  Sick sinus syndrome  Heart block and bundle branch block | Heart arrhythmias |
|  | Heart failure | Heart insufficiency |
|  | Stroke and transient ischaemic attack | Stroke |
|  | Chronic obstructive pulmonary disease | COPD |
|  | Inflammatory arthritis  Osteoarthritis | Arthritis |
|  | Depression | Depression |
| **Elixhauser (1998)[4]** | **28 conditions**  HIV | AIDS/HIV |
|  | Aplastic anaemia  Sickle cell disease | Anaemia deficiency |
|  | Inflammatory arthritis | Rheumatoid arthritis |
|  | .. | Blood loss anaemia |
|  | Atrial fibrillation  Paroxysmal tachycardias  Sick sinus syndrome  Heart block and bundle branch block | Cardiac arrhythmia |
|  | Heart failure | Congestive heart failure |
|  | Chronic obstructive pulmonary disease | Chronic Pulmonary Disease |
|  | .. | Coagulopathy |
|  | Diabetes – other  Type 1 diabetes mellitus  Type 2 diabetes mellitus | Diabetes without Chronic Complications  Diabetes with Chronic Complications |
|  | Substance misuse | Drug Abuse |
|  | Hypertension | Hypertension |
|  | Thyroid disease | Hypothyroidism |
|  | Liver disease | Liver Disease |
|  | Haematological malignancy | Lymphoma |
|  | .. | Fluid and Electrolyte Disorders |
|  | Secondary malignancy | Metastatic Cancer |
|  | .. | Other Neurological Disorders |
|  | .. | Obesity |
|  | Paralysis | Paralysis |
|  | Peripheral arterial disease | Peripheral Vascular Disease |
|  | Primary pulmonary hypertension | Pulmonary Circulation Disorder |
|  | Chronic kidney disease | Renal Failure |
|  | Primary malignancy | Solid tumour without metastasis |
|  | Upper GASTRO-INTESTINAL acid disorders | Peptic ulcer disease |
|  | Heart valve disease | Valvular Disease |
|  | .. | Weight loss |
|  | Alcohol misuse | Alcohol abuse |
|  | Depression | Depression |
|  | .. | Psychoses |
| **Fortin (2017)[5]** | **27 conditions**  Hypertension | Hypertension |
|  | .. | Chronic musculoskeletal conditions causing pain |
|  | Inflammatory arthritis  Osteoarthritis | Arthritis and / or rheumatoid arthritis |
|  | Osteoporosis | Osteoporosis |
|  | Asthma  Chronic obstructive pulmonary disease | Asthma, chronic obstructive pulmonary disease (COPD), or chronic bronchitis |
|  | Coronary heart disease  Peripheral arterial disease | Cardiovascular disease (angina, myocardial infarction, atrial fibrillation, poor circulation in the lower limbs) |
|  | Heart failure | Heart failure (including valve problems or replacement) |
|  | Stroke and transient ischaemic attack | Stroke and transient ischaemic attack |
|  | Upper GASTRO-INTESTINAL acid disorders | Stomach problem (reflux, heartburn, or gastric ulcer) |
|  | Inflammatory bowel disease  Coeliac disease  Diverticulosis | Colon problems (irritable bowel, Crohn’s disease, ulcerative colitis, diverticulosis) |
|  | Liver disease | Chronic hepatitis |
|  | Diabetes – other  Type 1 diabetes mellitus  Type 2 diabetes mellitus | Diabetes |
|  | Thyroid disease | Thyroid disorder |
|  | Primary malignancy  Haematological malignancy  Secondary malignancy | Any cancer in the previous 5 years (including melanoma, but excluding other skin cancers) |
|  | Chronic kidney disease | Kidney disease or failure |
|  | Urinary incontinence | Chronic urinary problems |
|  | .. | Hyperlipidaemia (high cholesterol) |
|  | .. | Obesity (diagnosed through the calculation of body mass index) |
|  | Dementia | Dementia or Alzheimer’s disease |
|  | Depression  Anxiety | Depression or anxiety |
| **Ho always (2022)[6]** | **27 conditions**  Stroke and transient ischaemic attack | Stroke |
|  | Coronary heart disease | Coronary artery disease |
|  | Heart failure | Heart failure |
|  | Peripheral arterial disease | Peripheral artery disease |
|  | Diabetes – other  Type 1 diabetes mellitus  Type 2 diabetes mellitus | Diabetes |
|  | Addison’s disease | Addison’s disease |
|  | Cystic fibrosis | Cystic fibrosis |
|  | Chronic obstructive pulmonary disease | Chronic obstructive pulmonary disease |
|  | Asthma | Asthma |
|  | Parkinson’s disease | Parkinson’s disease |
|  | Epilepsy | Epilepsy |
|  | Multiple sclerosis | Multiple sclerosis |
|  | Paralysis | Paralysis |
|  | Primary malignancy | Solid organ cancers |
|  | Haematological malignancy | Haematological cancers |
|  | Secondary malignancy | Metastatic cancers |
|  | Lupus erythematosus  Sjogren syndrome  Scleroderma | Connective tissue disease |
|  | Liver disease | Chronic liver disease |
|  | Inflammatory bowel disease | Inflammatory bowel disease |
|  | Chronic kidney disease | Chronic kidney disease  End-stage kidney disease |
|  | HIV | HIV/AIDS |
|  | Dementia | Dementia |
|  | Schizophrenia | Schizophrenia |
| **Ho always + usually (2022)[6]** | **55 conditions**  Stroke and transient ischaemic attack | Stroke  Transient ischaemic attack |
|  | Coronary heart disease | Coronary artery disease |
|  | Heart failure | Heart failure |
|  | Peripheral arterial disease | Peripheral artery disease |
|  | Diabetes – other  Type 1 diabetes mellitus  Type 2 diabetes mellitus | Diabetes |
|  | Addison’s disease | Addison’s disease |
|  | Cystic fibrosis | Cystic fibrosis |
|  | Chronic obstructive pulmonary disease | Chronic obstructive pulmonary disease |
|  | Asthma | Asthma |
|  | Parkinson’s disease | Parkinson’s disease |
|  | Epilepsy | Epilepsy |
|  | Multiple sclerosis | Multiple sclerosis |
|  | Paralysis | Paralysis |
|  | Primary malignancy | Solid organ cancers  Melanoma |
|  | Haematological malignancy | Haematological cancers |
|  | Secondary malignancy | Metastatic cancers |
|  | Lupus erythematosus  Sjogren syndrome  Scleroderma | Connective tissue disease |
|  | Liver disease | Chronic liver disease |
|  | Inflammatory bowel disease | Inflammatory bowel disease |
|  | Chronic kidney disease | Chronic kidney disease  End-stage kidney disease |
|  | HIV | HIV/AIDS |
|  | Dementia | Dementia |
|  | Schizophrenia | Schizophrenia |
|  | Heart valve disease | Heart valve disorders |
|  | Atrial fibrillation  Paroxysmal tachycardias  Sick sinus syndrome  Heart block and bundle branch block | Arrythmia |
|  | Venous thromboembolic disease | Venous thromboembolic disease |
|  | .. | Aneurysm |
|  | Hypertension | Hypertension (treated and untreated) |
|  | Thyroid disease | Thyroid disorders |
|  | Bronchiectasis | Bronchiectasis |
|  | Peripheral neuropathy | Peripheral neuropathy |
|  | .. | Chronic primary pain |
|  | .. | Benign cerebral tumours that can cause disability |
|  | Osteoarthritis | Osteoarthritis |
|  | .. | Long-term musculoskeletal problems due to injury |
|  | Osteoporosis | Osteoporosis |
|  | Gout | Gout |
|  | .. | Chronic pancreatic disease |
|  | Upper GASTRO-INTESTINAL acid disorders | Peptic ulcer |
|  | Endometriosis | Endometriosis |
|  | .. | Chronic urinary tract infection |
|  | Aplastic anaemia  Sickle cell disease | Anaemia (including pernicious anaemia, sickle cell anaemia) |
|  | Visual impairment | Vision impairment that cannot be corrected |
|  | Hearing impairment | Hearing impairment that cannot be corrected |
|  | .. | Meniere’s disease |
|  | .. | Chronic Lyme disease |
|  | .. | Tuberculosis |
|  | .. | Post-acute covid-19 |
|  | Intellectual disability | Congenital disease and chromosomal abnormalities |
|  | Depression | Depression |
|  | Anxiety | Anxiety |
|  | Bipolar affective disorder | Bipolar disorder |
|  | Alcohol misuse  Substance misuse | Drug/alcohol misuse |
|  | Eating disorder | Eating disorder |
|  | Autism | Autism |
|  | .. | Post-traumatic stress disorder |
| **N’Goran (2016)[7]** | **29 conditions**  .. | Pain general / multiple sites |
|  | Primary malignancy | Malignancy not otherwise specified  Malignant neoplasm of stomach  Malignant neoplasm of colon/rectum  Malignancy neoplasm of pancreas  Malignant neoplasms of digest other / not otherwise specified  Malignant neoplasm thyroid  Malignant neoplasm nervous system  Malignant neoplasm bronchus / lung |
|  |  |  |
|  |  |  |
|  |  |  |
|  |  |  |
|  |  |  |
|  |  |  |
|  |  |  |
|  | .. | Secondary effect of trauma |
|  | Human immunodeficiency virus | HIV-infection / AIDS |
|  | Haematological malignancy | Hodgkin’s disease / lymphoma  Malignant neoplasm blood other |
|  |  |  |
|  | .. | Incontinence of bowel |
|  | .. | Irritable bowel syndrome |
|  | Inflammatory bowel disease | Chronic enteritis / ulcerative colitis |
|  | .. | Obesity |
|  | Type 1 diabetes mellitus | Diabetes insulin dependent |
|  | Type 2 diabetes mellitus | Diabetes non-insulin dependent |
|  | Gout | Gout |
|  | .. | Poliomyelitis |
|  | Multiple sclerosis | Multiple sclerosis |
|  | Parkinson’s disease | Parkinsonism |
|  | Epilepsy | Epilepsy |
|  | .. | Migraine |
|  | .. | Trigeminal neuralgia |
|  | .. | Abnormal involuntary movements |
|  | Peripheral neuropathy | Peripheral neuritis / neuropathy |
|  | .. | Pain face |
|  | .. | Chronic ulcer skin |
|  | Inflammatory arthritis | Rheumatoid / seropositive arthritis |
|  | Chronic obstructive pulmonary disease | Chronic bronchitis |
|  | Chronic obstructive pulmonary disease | Chronic obstructive pulmonary disease |
|  | Asthma | Asthma |
|  | .. | Retinopathy |
|  | .. | Macular degeneration |
|  | Visual impairment and blindness | Blindness |
|  | Hearing loss | Hearing complaint  Deafness |
|  |  |  |
|  | .. | Risk factor for cardiovascular disease |
|  | Ischaemic heart disease | Ischaemic heart disease with angina  Ischaemic heart disease without angina |
|  |  |  |
|  | Atrial fibrillation | Atrial fibrillation / flutter |
|  | Primary pulmonary hypertension | Pulmonary heart disease |
|  | Hypertension | Elevated blood pressure  Hypertension uncomplicated  Hypertension complicated |
|  |  |  |
|  |  |  |
|  | Stroke and transient ischaemic attack | Cerebrovascular disease |
|  | Peripheral arterial disease | Atherosclerosis / peripheral vascular disease |
|  | Alcohol misuse | Chronic alcohol abuse |
|  | .. | Tobacco abuse |
|  | Substance misuse | Drug abuse |
|  | Dementia | Dementia |
|  | Schizophrenia | Organic psychosis disorder |
|  | .. | Phobia / compulsive disorder |
|  | .. | Post-traumatic stress disorder |
|  | Intellectual disability | Mental retardation |
|  | Eating disorders | Anorexia nervosa / bulimia |
| **Salive (2013)[8]** | **19 conditions**  Hypertension | Hypertension |
|  | .. | Hyperlipidaemia |
|  | Coronary heart disease | Ischaemic heart disease |
|  | Diabetes – other  Type 1 diabetes mellitus  Type 2 diabetes mellitus | Diabetes |
|  | Inflammatory arthritis  Osteoarthritis | Arthritis |
|  | Heart failure | Heart failure |
|  | Chronic kidney disease | Chronic kidney disease |
|  | Osteoporosis | Osteoporosis |
|  | Chronic obstructive pulmonary disease | Chronic obstructive pulmonary disease |
|  | Atrial fibrillation | Atrial fibrillation |
|  | Primary malignancy  Haematological malignancy  Secondary malignancy | Cancer |
|  | Asthma | Asthma |
|  | Stroke and transient ischaemic attack | Stroke |
|  | Depression | Depression |
|  | Dementia | Alzheimer’s disease |

.. = No similar condition matched.

References:

1. Barnett K, Mercer SW, Norbury M, Watt G, Wyke S, Guthrie B. Epidemiology of multimorbidity and implications for health care, research, and medical education: a cross-sectional study. Lancet. 2012;380(9836):37-43.

2. Charlson ME, Pompei P, Ales KL, MacKenzie CR. A new method of classifying prognostic comorbidity in longitudinal studies: Development and validation. J Chronic Dis. 1987;40(5):373-83.

3. Diederichs C, Berger K, Bartels DB. The measurement of multiple chronic diseases--a systematic review on existing multimorbidity indices. J Gerontol A Biol Sci Med Sci. 2011;66(3):301-11.

4. Elixhauser A, Steiner C, Harris DR, Coffey RM. Comorbidity Measures for Use with Administrative Data. Medical care. 1998;36(1):8-27.

5. Fortin M, Almirall J, Nicholson K. Development of a Research Tool to Document Self-Reported Chronic Conditions in Primary Care. J Comorb. 2017;7(1):117-23.

6. Ho ISS, Azoaga-Lorenzo A, Akbari A, Davies J, Khunti K, Kadam U, et al Measuring multimorbidity in research: a Delphi consensus study. BMJ Medicine. July 2022.

7. N'Goran AA, Blaser J, Deruaz-Luyet A, Senn N, Frey P, Haller DM, et al. From chronic conditions to relevance in multimorbidity: A four-step study in family medicine. Fam Pract. 2016;33(4):439-44.

8. Salive ME. Multimorbidity in older adults. Epidemiol Rev. 2013;35(1):75-83.
