# Supplementary material for: The impact of varying the number and selection of conditions on estimated multimorbidity prevalence: A cross-sectional study using a large, primary care population dataset
Source: PLoS Med. 2023 Apr 4;20(4):e1004208. doi: 10.1371/journal.pmed.1004208 (PMC10072475; doi:10.1371/journal.pmed.1004208)
Supplement: S3 Table — Checklist of items, extended from the STROBE statement, that should be reported in observational studies using routinely collected health data. (DOCX) [file pmed.1004208.s003.docx]

# Supplementary Table 3. The RECORD statement.

Checklist of items, extended from the STROBE statement, that should be reported in observational studies using routinely collected health data.

|  | **Item No.** | **STROBE items** | **Location in manuscript where items are reported** | **RECORD items** | **Location in manuscript where items are reported** |
| --- | --- | --- | --- | --- | --- |
| **Title and abstract** | | | | | |
|  | 1 | (a) Indicate the study’s design with a commonly used term in the title or the abstract (b) Provide in the abstract an informative and balanced summary of what was done and what was found | **Title and abstract.**  **The study was a cross-sectional cohort study, and this is included in the abstract and methods sections.** | RECORD 1.1: The type of data used should be specified in the title or abstract. When possible, the name of the databases used should be included.  RECORD 1.2: If applicable, the geographic region and timeframe within which the study took place should be reported in the title or abstract.  RECORD 1.3: If linkage between databases was conducted for the study, this should be clearly stated in the title or abstract. | - 1. **Methods section of the abstract. The database name is used in the methods section of the main article.**   2. **Methods section of the abstract and main article.**   3. **Not applicable.** |
| **Introduction** | | | | | |
| Background rationale | 2 | Explain the scientific background and rationale for the investigation being reported | **“Background” section of the abstract, and “Introduction” section of the main article**. |  |  |
| Objectives | 3 | State specific objectives, including any prespecified hypotheses | **Final paragraph of the “Background” section of the abstract, and “Introduction” section of the main article.** |  |  |
| **Methods** | | | | | |
| Study Design | 4 | Present key elements of study design early in the paper | **“Study design” section of “Methods” in the main article.** |  |  |
| Setting | 5 | Describe the setting, locations, and relevant dates, including periods of recruitment, exposure, follow-up, and data collection | **“Methods” section of the abstract, and “Statistical analysis” section of “Methods” in the main article.** |  |  |
| Participants | 6 | *(a) Cohort study* - Give the eligibility criteria, and the sources and methods of selection of participants. Describe methods of follow-up  *Case-control study* - Give the eligibility criteria, and the sources and methods of case ascertainment and control selection. Give the rationale for the choice of cases and controls  *Cross-sectional study* - Give the eligibility criteria, and the sources and methods of selection of participants  *(b) Cohort study* - For matched studies, give matching criteria and number of exposed and unexposed  *Case-control study* - For matched studies, give matching criteria and the number of controls per case | **“Methods” section of the abstract, and “Results” section of the main article.** | RECORD 6.1: The methods of study population selection (such as codes or algorithms used to identify subjects) should be listed in detail. If this is not possible, an explanation should be provided.  RECORD 6.2: Any validation studies of the codes or algorithms used to select the population should be referenced. If validation was conducted for this study and not published elsewhere, detailed methods and results should be provided.  RECORD 6.3: If the study involved linkage of databases, consider use of a flow diagram or other graphical display to demonstrate the data linkage process, including the number of individuals with linked data at each stage. | **6.1 “Definition of variables” in “Methods” section of the main article explained the methodology we used to develop clinical code lists.**  **6.2 Code lists are available from the HDR UK Phenotype Library.**  **6.3 No data linkage was required.** |
| Variables | 7 | Clearly define all outcomes, exposures, predictors, potential confounders, and effect modifiers. Give diagnostic criteria, if applicable. | **“Statistical analysis” section of “Methods” in the main article.** | RECORD 7.1: A complete list of codes and algorithms used to classify exposures, outcomes, confounders, and effect modifiers should be provided. If these cannot be reported, an explanation should be provided. | **All code lists are available on the HDR UK Phenotype Library and listed in the supplementary material.** |
| Data sources/ measurement | 8 | For each variable of interest, give sources of data and details of methods of assessment (measurement).  Describe comparability of assessment methods if there is more than one group | **“Data sources” and “Statistical analysis” sections of “Methods” in the main article.** |  |  |
| Bias | 9 | Describe any efforts to address potential sources of bias | **“Data sources” section of “Methods” in the main article reports how GP electronic health records, hospital admission data, and laboratory results were all used to define morbidities to combat bias where clinicians were not recording diagnoses.** |  |  |
| Study size | 10 | Explain how the study size was arrived at | **“Study participants” section of “Methods” in the main article. The entire population alive and registered with the 149 general practices at the study cross-section date were included. There was no need to take a sample from the population.** |  |  |
| Quantitative variables | 11 | Explain how quantitative variables were handled in the analyses. If applicable, describe which groupings were chosen, and why | **Not applicable.** |  |  |
| Statistical methods | 12 | (a) Describe all statistical methods, including those used to control for confounding  (b) Describe any methods used to examine subgroups and interactions  (c) Explain how missing data were addressed  (d) *Cohort study* - If applicable, explain how loss to follow-up was addressed  *Case-control study* - If applicable, explain how matching of cases and controls was addressed  *Cross-sectional study* - If applicable, describe analytical methods taking account of sampling strategy  (e) Describe any sensitivity analyses | **“Statistical analysis” section of “Methods” in the main article.** |  |  |
| Data access and cleaning methods |  | .. |  | RECORD 12.1: Authors should describe the extent to which the investigators had access to the database population used to create the study population.  RECORD 12.2: Authors should provide information on the data cleaning methods used in the study.12.1 | **12.1 The last line of acknowledgements.**  **12.2 Minimal data cleaning was required due to the nature of the study.** |
| Linkage |  | .. |  | RECORD 12.3: State whether the study included person-level, institutional-level, or other data linkage across two or more databases. The methods of linkage and methods of linkage quality evaluation should be provided. | **No linkage was required.** |
| **Results** | | | | | |
| Participants | 13 | (a) Report the numbers of individuals at each stage of the study (*e.g.*, numbers potentially eligible, examined for eligibility, confirmed eligible, included in the study, completing follow-up, and analysed)  (b) Give reasons for non-participation at each stage.  (c) Consider use of a flow diagram | **Not required as the whole population of the 149 general practices was included.** | RECORD 13.1: Describe in detail the selection of the persons included in the study (*i.e.,* study population selection) including filtering based on data quality, data availability and linkage. The selection of included persons can be described in the text and/or by means of the study flow diagram. | **Not applicable as the whole population was included. However, this is discussed in “Study participants” section of “Methods” in the main article.** |
| Descriptive data | 14 | (a) Give characteristics of study participants (*e.g.*, demographic, clinical, social) and information on exposures and potential confounders  (b) Indicate the number of participants with missing data for each variable of interest  (c) *Cohort study* - summarise follow-up time (*e.g.*, average and total amount) | **Table 1 main article. No data were missing.** |  |  |
| Outcome data | 15 | *Cohort study* - Report numbers of outcome events or summary measures over time  *Case-control study* - Report numbers in each exposure category, or summary measures of exposure  *Cross-sectional study* - Report numbers of outcome events or summary measures | **Figures 1, 2, 3, 4, and 5 in the main article.** |  |  |
| Main results | 16 | (a) Give unadjusted estimates and, if applicable, confounder-adjusted estimates and their precision (e.g., 95% confidence interval). Make clear which confounders were adjusted for and why they were included  (b) Report category boundaries when continuous variables were categorized  (c) If relevant, consider translating estimates of relative risk into absolute risk for a meaningful time period | **“Results” section of the main article.** |  |  |
| Other analyses | 17 | Report other analyses done—e.g., analyses of subgroups and interactions, and sensitivity analyses | **None undertaken.** |  |  |
| **Discussion** | | | | | |
| Key results | 18 | Summarise key results with reference to study objectives | **“Key findings” section and first paragraph of “Discussion” section of the main article.** |  |  |
| Limitations | 19 | Discuss limitations of the study, taking into account sources of potential bias or imprecision. Discuss both direction and magnitude of any potential bias | **“Discussion” section of the main article.** | RECORD 19.1: Discuss the implications of using data that were not created or collected to answer the specific research question(s). Include discussion of misclassification bias, unmeasured confounding, missing data, and changing eligibility over time, as they pertain to the study being reported. | **Second section of the third paragraph in the “Discussion” section of the main article.** |
| Interpretation | 20 | Give a cautious overall interpretation of results considering objectives, limitations, multiplicity of analyses, results from similar studies, and other relevant evidence | **“Discussion” section of the main article.** |  |  |
| Generalisability | 21 | Discuss the generalisability (external validity) of the study results | **“Discussion” section of the main article.** |  |  |
| **Other Information** | | | | | |
| Funding | 22 | Give the source of funding and the role of the funders for the present study and, if applicable, for the original study on which the present article is based | **“Funding” section in the abstract.** |  |  |
| Accessibility of protocol, raw data, and programming code |  | .. |  | RECORD 22.1: Authors should provide information on how to access any supplemental information such as the study protocol, raw data, or programming code. | **Available in supplementary material.** |

*Reference: Benchimol EI, Smeeth L, Guttmann A, Harron K, Moher D, Petersen I, Sørensen HT, von Elm E, Langan SM, the RECORD Working Committee. The REporting of studies Conducted using Observational Routinely-collected health Data (RECORD) Statement. *PLoS Medicine* 2015; in press.
