# Supplementary material for: The impact of varying the number and selection of conditions on estimated multimorbidity prevalence: A cross-sectional study using a large, primary care population dataset
Source: PLoS Med. 2023 Apr 4;20(4):e1004208. doi: 10.1371/journal.pmed.1004208 (PMC10072475; doi:10.1371/journal.pmed.1004208)
Supplement: Attachment — Submitted filename: PLOS_Medicine_reviewer_response_Jan_2023.docx [file pmed.1004208.s007.docx]

Dear Dr. MacRae,

Thank you very much for submitting your manuscript "Measuring multimorbidity: impact of varying the number and selection of conditions on estimated multimorbidity prevalence in a large primary care population dataset" (PMEDICINE-D-22-03375R1) for consideration at PLOS Medicine.

Your paper was evaluated by a senior editor and discussed among all the editors here. It was also discussed with an academic editor with relevant expertise, and sent to independent reviewers, including a statistical reviewer. The reviews are appended at the bottom of this email and any accompanying reviewer attachments can be seen via the link below:


In light of these reviews, I am afraid that we will not be able to accept the manuscript for publication in the journal in its current form, but we would like to consider a revised version that addresses the reviewers' and editors' comments. Obviously we cannot make any decision about publication until we have seen the revised manuscript and your response, and we plan to seek re-review by one or more of the reviewers.

In revising the manuscript for further consideration, your revisions should address the specific points made by each reviewer and the editors. Please also check the guidelines for revised papers at <http://journals.plos.org/plosmedicine/s/revising-your-manuscript> for any that apply to your paper. In your rebuttal letter you should indicate your response to the reviewers' and editors' comments, the changes you have made in the manuscript, and include either an excerpt of the revised text or the location (eg: page and line number) where each change can be found. Please submit a clean version of the paper as the main article file; a version with changes marked should be uploaded as a marked up manuscript.

In addition, we request that you upload any figures associated with your paper as individual TIF or EPS files with 300dpi resolution at resubmission; please read our figure guidelines for more information on our requirements: <http://journals.plos.org/plosmedicine/s/figures>. While revising your submission, please upload your figure files to the PACE digital diagnostic tool, <https://pacev2.apexcovantage.com/>. PACE helps ensure that figures meet PLOS requirements. To use PACE, you must first register as a user. Then, login and navigate to the UPLOAD tab, where you will find detailed instructions on how to use the tool. If you encounter any issues or have any questions when using PACE, please email us at PLOSMedicine@plos.org.

We expect to receive your revised manuscript by Jan 04 2023 11:59PM. Please email us (plosmedicine@plos.org) if you have any questions or concerns.

***Please note while forming your response, if your article is accepted, you may have the opportunity to make the peer review history publicly available. The record will include editor decision letters (with reviews) and your responses to reviewer comments. If eligible, we will contact you to opt in or out.***

We ask every co-author listed on the manuscript to fill in a contributing author statement, making sure to declare all competing interests. If any of the co-authors have not filled in the statement, we will remind them to do so when the paper is revised. If all statements are not completed in a timely fashion this could hold up the re-review process. If new competing interests are declared later in the revision process, this may also hold up the submission. Should there be a problem getting one of your co-authors to fill in a statement we will be in contact.  YOU MUST NOT ADD OR REMOVE AUTHORS UNLESS YOU HAVE ALERTED THE EDITOR HANDLING THE MANUSCRIPT TO THE CHANGE AND THEY SPECIFICALLY HAVE AGREED TO IT. You can see our competing interests policy here: <http://journals.plos.org/plosmedicine/s/competing-interests>.

Please use the following link to submit the revised manuscript:

<https://www.editorialmanager.com/pmedicine/>

Your article can be found in the "Submissions Needing Revision" folder.

To enhance the reproducibility of your results, we recommend that you deposit your laboratory protocols in protocols.io, where a protocol can be assigned its own identifier (DOI) such that it can be cited independently in the future. Additionally, PLOS ONE offers an option to publish peer-reviewed clinical study protocols. Read more information on sharing protocols at <https://plos.org/protocols?utm_medium=editorial-email&utm_source=authorletters&utm_campaign=protocols>

Please ensure that the paper adheres to the PLOS Data Availability Policy (see <http://journals.plos.org/plosmedicine/s/data-availability)>, which requires that all data underlying the study's findings be provided in a repository or as Supporting Information. For data residing with a third party, authors are required to provide instructions with contact information for obtaining the data. PLOS journals do not allow statements supported by "data not shown" or "unpublished results." For such statements, authors must provide supporting data or cite public sources that include it.

We look forward to receiving your revised manuscript.

Sincerely,

Philippa Dodd, MBBS MRCP PhD
PLOS Medicine
plosmedicine.org


-----------------------------------------------------------
Requests from the editors:

GENERAL
Please respond to all editor and reviewer requests detailed below, in full.

**Response 1.** Thank you very much for the opportunity to provide changes to this manuscript. Please find responses with amended text for each request below. Changed text is represented by bold text in the responses below and as tracked changes in the marked-up document attached.

Please number the lines in the manuscript starting at 1 at “Abstract” and in continuous sequence thereafter.

**Response 2**. Please find continuous line numbering of main text.

We agree with the reviewers (please see below) that the study’s primary goal should be better emphasized/identified and its implications.  Further details and clarifications are required throughout, which we agree with.  Please revise accordingly.

** We agree that the novelty is a bit lost among the formal data reporting.  Please revise in mind of the potential impact of the study outcomes at the patient/policy level **

**Response 3.** Please see changes made throughout the abstract and main text to make the novel findings and potential impact more prominent.

COMMENTS FROM THE ACADEMIC EDITOR
I reviewed the article and the reviews and I agree with the decision for major revision

ABSTRACT
Please structure your abstract using the PLOS Medicine headings (Background, Methods and Findings, Conclusions). Please combine the Methods and Findings sections into one section, “Methods and findings”.

**Response 4.** Please see revised abstract headings on page 2 line 30 and page 3 line 50.

Abstract Methods and Findings:
Please ensure that all numbers presented in the abstract are present and identical to numbers presented in the main manuscript text.

**Response 5.** All numbers presented in abstract, main text, and supplementary file have been checked and are identical.

Please provide further details on the population (and setting) that the study investigates

**Response 6.** Please see addition to Abstract Methods and Findings section on page 2 lines 31-32.

“Cross-sectional study using English primary care data for 1168260 participants **who were all people alive and permanently registered with 149 included general practices**.”

Please provide further details as to how conditions were “selected” when considering multimorbidity prevalence (diagnostic coded registers?)

**Response 7.** Please see addition to Abstract Methods and Findings section on page 2 lines 33-35.

“**for 80 conditions featured in phenotyping algorithms in the HDR-UK Phenotype Library and/or in ≥one of the nine published lists of conditions examined in the study.**”

Please clearly define the main outcome measures.

**Response 8.** Please see addition to Abstract Methods and Findings section on page 2 line 32.

“**Outcome measures of the study were prevalence estimates of multimorbidity** **when** varying the number and selection of conditions considered (≥two conditions)”

Please quantify the main results with p-values as well as 95% CIs, if not for the purpose of transparent data reporting please provide the reasons why

**Response 9.** Please see addition of p-values to the 95% confidence intervals to Abstract Methods and Findings and Main text Results. section on page 2 lines 39-41.

Please include any important dependent variables that are adjusted for in the analyses.

**Response 10.** Please see addition to Abstract Methods and Findings section on page 2 line 38.

“Analyses were stratified by **dependent variables** age, socioeconomic position, and sex”

Please include a summary of adverse events if these were assessed in the study.

**Response 11.** No adverse events were assessed in the study.

In the last sentence of the Abstract Methods and Findings section, please describe the main limitation(s) of the study's methodology.

**Response 12.** Please see addition to Abstract Methods and Findings section on page 2 lines 45-48.

“**A limitation of the study is that conditions were not always replicated using the same ascertainment rules as previous studies to improve comparability across condition lists, but this highlights further variability in prevalence estimates across studies.”**

Abstract Conclusions:
Please replace the sub-heading “interpretation” with “conclusions”

**Response 13.** Please see replacement term in Abstract Conclusion section on page 3 line 50.

Please address the study implications without overreaching what can be concluded from the data; the phrase "In this study, we observed ..." may be useful.

**Response 14.** Please see addition to Abstract Conclusion section on page 3 line 50.

“**In this study we observed that v**arying the number and selection of conditions results in very large differences in multimorbidity prevalence.”

Please interpret the study based on the results presented in the abstract, emphasizing what is new and what the wider impact of the study outcomes are, without overstating your conclusions.

Please avoid vague statements such as "these results have major implications for policy/clinical care".  Mention only specific implications substantiated by the results.
Please avoid assertions of primacy ("We report for the first time....")

**Response 15.** Please see addition to Abstract Conclusion section on page 3 lines 50-55.

**“In this study we observed that v**arying the number and selection of conditions results in very large differences in multimorbidity prevalence**, and different numbers of conditions are needed to reach ceiling rates of multimorbidity prevalence in certain groups of people. These findings imply that there is a need for standardisation and to address this, researchers can consider using the condition-lists identified in this study resulting in the highest multimorbidity prevalence: Ho always + usually, Barnett, or Fortin.”**

AUTHOR SUMMARY
At this stage, we ask that you include a short, non-technical Author Summary of your research to make findings accessible to a wide audience that includes both scientists and non-scientists. The Author Summary should immediately follow the Abstract in your revised manuscript. This text is subject to editorial change and should be distinct from the scientific abstract. Please see our author guidelines for more information: <https://journals.plos.org/plosmedicine/s/revising-your-manuscript#loc-author-summary>

**Response 16.** Please see addition of the author summary on page 4 lines 59-90.

“**Author summary**

**Why was this study done?**

- **There is wide variety in the conditions considered by researchers when measuring multimorbidity prevalence.**
- **A systematic review of 566 studies, published in 2021, found lack of consensus in the selection of conditions considered; in half of studies only eight conditions (diabetes, stroke, cancer, chronic obstructive pulmonary disease, hypertension, coronary heart disease, chronic kidney disease, and heart failure) were consistently considered; and the number of conditions considered varied from two to 285 (median 17).**
- **A more consistent approach to measuring multimorbidity is needed.**

**What did the researchers do and find?**

- **This study investigated the relationship between the number and selection of conditions considered and multimorbidity prevalence.**
- **People who are the oldest, living in the most deprived areas, and men require fewer conditions to be considered to reach close to multimorbidity prevalence when considering all 80 conditions (the ceiling effect, where the prevalence approaches the upper limit of prevalence possible in the study).**
- **There are large differences in prevalence when different numbers and selections of conditions are considered.**
- **Condition-lists resulting in highest multimorbidity prevalence were Ho always + usually, Barnett, and Fortin.**

**What do these findings mean?**

- **All conditions were counted in the same way (presence of the condition ever recorded) to improve comparability, however in previous studies conditions were counted according to varying rules, highlighting that further variability across studies will happen.**
- **There is a need for standardisation when measuring multimorbidity prevalence so that population subgroups are accurately represented**
- **To address this, researchers can consider using the Ho always + usually, Barnett, or Fortin condition lists.**”

INTRODUCTION
Please see reviewer comments – we agree that limitations to the age of the data-set should be discussed, please do so later in the discussion

**Response 17.** Please see addition to Main Text Discussion section on page 16 lines 359-362.

“**The data were from 2015 however prevalence of the commonest condition hypertension was unchanged between 1990 and 2019 in a pooled analysis of worldwide population studies (32% of women in 2019 versus 32% in 1990),[25] and this study identified similar prevalence rates of depression as ascertained by an Office of National Statistics survey from 2021.[26]”**

METHODS and RESULTS
Page 4, data sources: please define SEP at first use in this section

**Response 18.** Please see addition to Main Text Methods Data Sources section on page 8 line 150.

“**Socioeconomic position (**SEP**)** was defined as deciles of the English Index of Multiple Deprivation (IMD), a measure of relative deprivation according to small local area level, with deciles defined by national thresholds.”

Did your study have a prospective protocol or analysis plan? Please state this (either way) early in the Methods section.
a) If a prospective analysis plan (from your funding proposal, IRB or other ethics committee submission, study protocol, or other planning document written before analyzing the data) was used in designing the study, please include the relevant prospectively written document with your revised manuscript as a Supporting Information file to be published alongside your study, and cite it in the Methods section. A legend for this file should be included at the end of your manuscript.

**Response 19.** Please see addition to Main Text Methods Study design section on page 7 lines 135-137.

**“The analyses were designed in November 2021 and performed in February 2022, and no data-driven changes to analyses took place during this period.”**

b) If no such document exists, please make sure that the Methods section transparently describes when analyses were planned, and when/why any data-driven changes to analyses took place.
c) In either case, changes in the analysis-- including those made in response to peer review comments-- should be identified as such in the Methods section of the paper, with rationale.

**Response 20.** Please see addition to Main Text Methods Study design section on page 7 lines 137-140.

**“As part of the peer review process, one additional condition-list was added[11], and the paper updated to include p-values as well as confidence intervals for proportions, and sensitivity analyses of variation in prevalence by deprivation and gender using raw rather than direct age-standardised data were added.**”

Please see addition to Main Text Methods Statistical analyses section on page 10 line 216-219.

“Since age is very strongly associated with multimorbidity **and the SEP and sex composition within different age-groups varies making age a major confounder, analyses were standardised to the age-structure of the whole study cohort as the standard population and age-specific standardised rates for population subgroups were calculated.[20] Sensitivity analysis was done using unstandardised rates.**”

Please see addition to Main text Results section on page 13 lines 279-293.

**“In IMD-stratified analysis, there was a clear social gradient of multimorbidity prevalence irrespective of the number of conditions included, with the more deprived having higher prevalence than the less deprived (Figure 3).** The predefined ceiling was reached at a lower number of conditions in the most deprived IMD decile (49 conditions) compared to the least deprived (54 conditions) (Figure 3). **Sensitivity analysis using raw (unstandardised rates) had less clear social gradient (reflecting that the most deprived are on average younger than the affluent), and no clear pattern in the predefined ceiling across IMD deciles (Supplementary Figure 1).**

**Multimorbidity prevalence was higher in women and girls at every level of number of conditions in the direct age-standardised analysis, and the predefined ceiling was reached at a higher number of conditions in women and girls than in men and boys (Figure 4). In the sensitivity analysis using unstandardised rates, there was a larger gap in multimorbidity prevalence between sexes, reflecting that women are on average older, and the predefined ceiling was reached at a similar number of conditions to the direct age-standardised analysis (Supplementary Figure 2).**”

Please see addition to Figure 3 title and caption on page 31.

**“**Direct age standardisation where the whole study cohort was the standard population was applied **(see Supplementary Figure 1 for non-standardised rates).**”

For all observational studies, we ask authors to indicate the following clearly in the manuscript text:
(1) the specific hypotheses you intended to test,

**Response 21.** Please see page 7 line 133.

**“**A cross-sectional study design was used to examine **the hypothesis that** multimorbidity prevalence varie**s** when considering different numbers of conditions, and different selections of conditions (using published recommended or commonly used condition-lists), in the count”

(2) the analytical methods by which you planned to test them,
(3) the analyses you actually performed, and
(4) when reported analyses differ from those that were planned, transparent explanations for differences that affect the reliability of the study's results. If a reported analysis was performed based on an interesting but unanticipated pattern in the data, please be clear that the analysis was data-driven.

**Response 22.** See responses 19 and 20.

Please quantify the main results with p-values as well as 95% CIs, if not for the purpose of transparent data reporting please provide the reasons why
When a p value is given, please specify the statistical test used to determine it.

**Response 23.** Please see addition of p-values to all reported confidence intervals in main text.

Please see addition to Methods Statistical analysis on Page 10 lines 203-205.

“**The prop.test procedure in R was used to estimate 95% confidence intervals and p-values for prevalence using the normal approximation for large samples.**”

Please remove role of the funding source from the end of the methods section and include only in the manuscript submission form.

**Response 24.** Please see deletion to Methods Role of funding source on Page 11 line 228.

TABLES and FIGURES
We agree the reviewer that the figures rather undersell the data. The titles are rather inaccessible and difficult to understand and don’t explain the figure contents clearly for the reader. Please revise throughout, including the supporting files.
To make figures more accessible to those with colorblindness please consider avoiding the use of red/green
Please ensure that each figure/table (including those in supporting files) has an appropriate caption
Please define all abbreviations (for example: IMD, RR) in the figure/table captions
Please indicate whether analyses are adjusted and if so, please state  the caption in which factors are adjusted for and report unadjusted analyses for comparison

**Response 25.** Please see removal of all red and green from figures and use of a colourblind suitable palette in R.

Please see amendment and addition to Title and Caption for Figure 1. In Tables and Figures on page 26.

“Figure 1. Multimorbidity prevalence according to number **of conditions, the ceiling effect where adding additional conditions had little impact on prevalence, and** selection **of conditions using existing** condition-list**s**. **The black line represents multimorbidity prevalence calculated when considering different numbers of conditions in the count ranging from two to all 80 conditions, where conditions were added in order of most to least prevalent (e.g., at two conditions this is multimorbidity prevalence considering the most common two conditions). Percentage prevalence of multimorbidity when 10, 20, 30, 40, 50, 60, 70, and 80 conditions were considered marked at empty black circles above the black line. The number of conditions at which relative risk (RR) was >0.99 of multimorbidity prevalence of having the same multimorbidity prevalence when all 80 conditions were considered (ceiling effect) was reached is marked with an orange dot (at 52 conditions). Black dots represent multimorbidity prevalence when considering conditions included in existing condition-lists and are annotated with the condition-list name, prevalence, and number of conditions considered.**”

Please see amendment and addition to Title and Caption for Table 3. In Tables and Figures on page 28.

“Table 3. Multimorbidity prevalence **using existing** condition-list**s and** relative risk (RR) of multimorbidity when considering all 80 conditions **(ceiling effect where adding additional conditions had little impact on prevalence).**”

Please see amendment and addition to Title and Caption for Figure 2. In Tables and Figures on page 29.

**“**Figure 2. **Age-stratified multimorbidity prevalence according to number of conditions considered, reporting the ceiling effect where adding additional conditions had little impact on prevalence. Labelled coloured lines represent multimorbidity prevalence calculated when considering different numbers of conditions in the count ranging from two to all 80 conditions stratified into age groups. Black dots represent the number of conditions at which relative risk (RR) >0.99 of multimorbidity prevalence of having the same multimorbidity prevalence when all 80 conditions were considered (ceiling effect): 0-9 years at 71 conditions, 10-19 years at 67 conditions, 20-29 conditions at 57 conditions, 30-39 years at 57 conditions, 40-49 years at 56 conditions, 50-59 years at 50 conditions, 60-69 years at 44 conditions, 70-79 years at 37 conditions, 80+ years at 29 conditions.”**

Please see amendment and addition to Title and Caption for Figure 3. In Tables and Figures on page 31.

“Figure 3. **Socioeconomic position stratified multimorbidity prevalence according to number of conditions considered following direct age standardisation, reporting the ceiling effect where adding additional conditions had little impact on prevalence. Labelled coloured lines represent multimorbidity prevalence calculated when considering different numbers of conditions in the count ranging from two to all 80 conditions stratified into Index of Multiple Deprivation (IMD) deciles where IMD 1 is least and IMD 10 is most deprived. Black dots represent the number of conditions at which relative risk (RR) >0.99 of multimorbidity prevalence of having the same multimorbidity prevalence when all 80 conditions were considered (ceiling effect): IMD 10 at 49 conditions, IMD 9 at 50 conditions, IMD 8 at 50 conditions, IMD 7 at 51 conditions, IMD 6 at 51 conditions, IMD 5 at 53 conditions, IMD 4 at 52 conditions, IMD 3 at 53 conditions, IMD 2 at 53 conditions, and IMD 1 at 54 conditions. Direct age standardisation where the whole study cohort was the standard population was applied (see Supplementary Figure 1 for non-standardised rates).**”

Please see amendment and addition to Title and Caption for Figure 4. In Tables and Figures on page 33.

**“**Figure 4. **Sex stratified multimorbidity prevalence according to number of conditions considered following direct age standardisation, reporting the ceiling effect where adding additional conditions had little impact on prevalence.** Labelled coloured lines represent multimorbidity prevalence calculated when considering different numbers of conditions in the count ranging from two to all 80 conditions stratified by sex. Black dots represent the number of conditions at which relative risk (RR) >0.99 of multimorbidity prevalence of having the same multimorbidity prevalence when all 80 conditions were considered (ceiling effect): women and girls at 54 conditions and men and boys at 50 conditions. Direct age standardisation where the whole study cohort was the standard population was applied (see Supplementary Figure 2 for unstandardised rates).”

Please see amendment and addition to Title and Caption for Figure 5. In Tables and Figures on page 35.

“Figure **5. Multimorbidity prevalence by age considering all 80 conditions and according to existing condition-lists. Labelled coloured lines represent multimorbidity prevalence calculated for each age group when considering conditions in each condition-list.”**

DISCUSSION
Please present and organize the Discussion as follows: a short, clear summary of the article's findings; what the study adds to existing research and where and why the results may differ from previous research; strengths and limitations of the study; implications and next steps for research, clinical practice, and/or public policy; one-paragraph conclusion. Please remove the sub-heading conclusion.

**Response 26.** Please see deletion of sub heading “Conclusion” in Main Text Discussion section on page 18 line 401.

Please expand your discussion to better focus on the study aim and implication of the outcomes, without overstating – see also reviewer 2 comments

**Response 27.** Please see Main text Discussion on page 14 lines 312-315.

“This study found very large differences in estimated multimorbidity prevalence from varying the number and selection of conditions considered in the count**, and in younger people, the more affluent, and women, including additional relatively rare conditions had larger impact on estimated multimorbidity prevalence. M**ultimorbidity prevalence differed considerably by varying the **number of conditions, ranging from 4.6% to 40.5%, and** selection of conditions considered, ranging from 11.1% to 36.4% using **nine** previously published lists of conditions.[6, 9, 10, 16-19]”

Please remove all “declaration” statements from the end of the discussion and include only in the submission form.  The section “Author contributions” may remain under its own heading.

**Response 28.** Please see deletion of declarations except “Author contributions” on page 19.

REFERENCES
Please use the "Vancouver" style for reference formatting, and see our website for other reference guidelines here: <https://journals.plos.org/plosmedicine/s/submission-guidelines#loc-references>

For in-text reference call outs citations should be placed in square brackets and preceding punctuation like so [1,2,3,4] or [1-4,6].  Please amend throughout.

In the bibliography please list up to but no more than 6 author names followed by et al (where more than 6 authors contribute to the study).

Journal name abbreviations should be those found in the National Center for Biotechnology Information (NCBI) databases.

**Response 29.** Please see amended references on page 19 to include Vancouver square brackets, bibliography author lists, and journal names according to NCBI databases on page 20-21.

Comments from the reviewers:

Reviewer #1: Statistical review
This paper uses data from a large set of patients in England to investigate how different definitions of multimorbidity affect prevalence estimates. The authors also present this stratified by socioeconomic level, sex and age.

Generally the paper presents an important message for multimorbidity research. The statistical methods used are straightforward, as just prevalence estimates and confidence intervals are needed. I had very few comments, which are presented below:

1.      Statistical methods: "To examine the effect of the number of conditions considered in the count, multimorbidity prevalence was calculated when considering the most common two conditions, followed by the most common three conditions, the most common four conditions, etc, for every number up to considering all 80 conditions in the count (Information Panel). To do this, conditions were ordered from most to least prevalent (Supplementary Table 1) and added in turn to each new count" Although this procedure would give the highest multimorbidity prevalence in the case that conditions are independent from one another, it's possible it might not do so if common conditions are correlated. E.g. if considering conditions 1, 2 and 3 in descending prevalence, the prevalence of '1 and 2' might be less than '1 and 3' if conditions 1 and 2 co-occur in a lot of patients. This is just a picky point really and I don't think it makes sense to change what the authors did in any way!

**Response 30.** Thank you for making this important point. We agree that considering only two conditions in the count is unusual, however the recent systematic review by [Ho et al published in 2021](https://www.sciencedirect.com/science/article/pii/S2468266721001079?via%3Dihub#sec1) found that existing studies considered two to 285 conditions in a single multimorbidity measure, and considering the entire range of possibilities for this area with very little consensus is important to build understanding of how prevalence figures vary depending on the full range of possible choices. Therefore, we have left the analysis as examining two to 80 conditions, but we can change this to an alternative starting point if preferred.

2.      Statistical methods: can some more intuition be provided about the direct standardisation to the age structure of the population?

**Response 31.** Please see addition to Methods Statistical analyses section on page 10 line 216-219.

“Since age is very strongly associated with multimorbidity and **the SEP and sex composition within different age-groups varies making age a major confounder, analyses were standardised to the age-structure of the whole study cohort as the standard population and age-specific standardised rates for population subgroups were calculated.[20]**”

3.      Page 8: "29.5% (95%CI 29.5-29.9%)" - is this correct? The lower boundary and the point estimate are the same.

**Response 32.** Please see amendment of the upper bound to the 95% confidence interval at Main Text Results on page 11 line 245.

“29.5% (95%CI 29.5-29.**6**%, p <0.001)”

4.      Discussion: are the prevalence estimates generally an underestimate due to potential under-diagnosis of some conditions?

**Response 33.** Please see addition to Main Text Discussion section page 16 lines 356-359.

**“Using electronic health records to ascertain the prevalence of conditions can be associated with under ascertainment because the absence of a record does not necessarily mean absence of the condition, and more severe disease is likely to be over-represented in medical records.”**

James Wason

Reviewer #2: Thank you for the opportunity to review this work. In their manuscript, the authors explore how various definitions of multimorbidity influence its estimated prevalence. As the authors acknowledge, the growing attention to multimorbidity - and its highly varied operationalization - has contributed to methodological variation and biases in the literature. Overall, this manuscript covers an important aspect of multimorbidity research.

I have a few comments for the authors to consider. Overall, though, I think the paper would benefit from identifying the primary goal and really tightening up the methods, results, and discussion around this. It was, at times, a bit hard to follow. I am not advocating for removing any of the current analyses, but rather making sure they are presented in a way that easily conveys the overarching theme and story while highlighting the important and novel aspects of this work.

Major:

1.      Introduction: the authors state "it is common for authors not to explain the procedure used to decide which conditions they consider in the count" - I don't think this is necessarily true. Instead, there's often a 'recycling' of previously published lists. This could be reframed to highlight that the heterogeneity the authors are exploring is not due to authors not presenting information.

**Response 34.** Please see amendment and addition to Main Text Introduction section on page 6 lines 97-100.

“**Choice of conditions considered in the count (the denominator) when measuring multimorbidity prevalence is likely** to be driven by pragmatic decision making in the context of data availability**,[5] or by recycling of existing published condition lists,** and results in wide diversity in the number and selection of conditions considered in current multimorbidity literature.[6]”

2.      The authors acknowledge in the introduction that the relationship between multimorbidity and time is important to note given its growing prevalence. However, the data the authors analyze is now nearly 7 years old. While I doubt it would make any meaningful change in the results, the authors should consider acknowledging the datedness of these data and whether they believe this influences their findings at all.

**Response 35.** Please see **Response 17**.

3.      Would the authors consider making Supplemental Table 1 part of the main text? I think the list of included conditions is the crux of this study. I know it's visually unappealing, but I think could be important.

**Response 36.** Please see relocation to Main Text Results as Table 2 on pages 24-25.

4.      Could the authors, in the methods, more clearly state that they included every condition mention in any of those studies? It seems to me that is what the authors did, but I think this needs to be made explicitly clear.

**Response 37.** Please see rearrangement of text in Abstract Methods section on page 2 lines 33-35.

**“**for 80 conditions **featured in ≥one of the nine published lists of conditions examined in the study** and/or phenotyping algorithms in the HDR-UK Phenotype Library.

Please see rearrangement of text in Main Text Methods Definition of variables section on page 8 lines 166-168 and page 9 lines 169-170.

"The 80 conditions were chosen because they featured in **one or more of the nine published lists of conditions examined in the study and/or** phenotyping algorithms **(condition code lists)** in the HDR-UK Phenotype Library.[14] **New code lists were made by study authors for conditions featured in exiting condition-lists where no HDR-UK algorithms were available and are listed in Supplementary Table 1.”**

5.      The approach to use a relative risk and evaluate the appropriate 'cutoff' given those 80 conditions is an interesting approach but could be better setup in the introduction and the methods. It's a bit confusing, right now, the various comparisons that are being made. Indeed, I'd encourage the authors to consider an approach where they explicitly and succinctly lay out all their analyses. For example, "We conducted a suite of comparisons including: ____."

**Response 38.** Please see Main Text Methods section on page 10 lines 195-196.

**“We conducted a suite of comparisons including** examin**ation of** the effect of the number of conditions considered in the count **on** multimorbidity prevalence **by** considering the most common two conditions, followed by the most common three conditions, the most common four conditions, etc, for every number up to considering all 80 conditions in the count (Information Panel).”

6.      The relative risk analysis seems to be the most novel part of this studied but is not as prominently featured. I would encourage the authors to restructure their manuscript focusing on this if they also believe it's the most novel. Right now, some of the most interesting findings feel a bit buried in the analysis.

**Response 39.** Please see reordering of results in Abstract Methods and Findings page 2 lines 41-44 into a more prominent position.

“**The threshold number of conditions at which prevalence was >99% of prevalence measured when considering all 80 conditions was 52 for the whole population but was lower in older people (29 in >80 years) and higher in younger people (71 in 0–9-year-olds).”**

Please see reordering of results in Main Text Statistical analysis page 10 lines 207-212 into a more prominent position.

“**By making the assumption that multimorbidity prevalence estimated by considering all 80 conditions in the count was the true prevalence, we then calculated the number of conditions that had to be included to exceed a relative risk (RR) of 0.99 of this ‘true’ prevalence. This was done to estimate when a ceiling effect was present, where the prevalence approaches the upper limit of possible prevalence in the study and the point at which adding further conditions to the count had very little impact on multimorbidity prevalence**.”

a.      Similarly, it's unclear what the intermediary analyses contribute. For example, "When considering only the two conditions…" as presented in the results. While I understand this was a step along the way, I'm not sure that any study would ever pick only two conditions to estimate the prevalence of multimorbidity.

**Response 40.** Please see **Response 30**.

b.      Additionally, I think the first sentence of the discussion doesn't highlight the most important findings. Of course, when it's 2 versus 80 there is a large difference. There are several other findings that are better to highlight - for example the sentence beginning "Second, multimorbidity…"

**Response 41.** Please see deletion and restructure of Main Text Discussion on page 14 line 312-315.

“This study found very large differences in estimated multimorbidity prevalence from varying the number and selection of conditions considered in the count, **and in younger people, the more affluent, and women, including additional relatively rare conditions had larger impact on estimated multimorbidity prevalence.** **M**ultimorbidity prevalence differed considerably by varying the **number of conditions, ranging from 4.6% to 40.5%, and** selection of conditions considered, ranging from 11.1% to 36.4% using eight previously published lists of conditions.[6, 9, 10, 16-19] Counting multimorbidity prevalence using the eight existing condition-lists resulted in lower estimated prevalence than when considering the same number of the most common conditions, although the extent of this varied: Ho always + usually,[10] Fortin,[9] and Barnett[16] had the best performance.”

Minor:

1.      The authors could remove the date from the abstract methods.

**Response 42.** Please see deletion from Abstract Methods and Findings section on page 2 line 32.

“Cross-sectional study using English primary care data for 1168260 participants who were all people alive and permanently registered with 149 included general practices.”

2.      The abstract findings are hard to follow without the additional context presented in the full manuscript. I would encourage the authors to try to restructure their findings to make it more interpretable.

**Response 43.** Please see Abstract Methods and Findings on page 2 lines 39-44 where the sentence reporting prevalence by number of conditions has been shortened, and the threshold number of conditions results have been moved towards the top.

“Prevalence when only the two commonest conditions were considered was 4.6% (95%CI 4.6-4.6, *p*-value <0001), rising to 29.5% (95%CI 29.5-29.6, *p*-value <0001) considering the 10 commonest, 35.2% (95%CI 35.1-35.3, *p*-value <0001) considering the 20 commonest**, and 40.5% (95%CI 40.4-40.6, *p*-value <0001) when considering all 80 conditions.** **The threshold number of conditions at which prevalence was >99% of prevalence measured when considering all 80 conditions was 52 for the whole population but was lower in older people (29 in >80 years) and higher in younger people (71 in 0–9-year-olds).** Using the nine published condition-lists, multimorbidity prevalence varied from 11.1% to 36.4%.”

3.      Could the authors instead spell out "November 30, 2015" since DD/MM/YY is not standard globally.

**Response 44.** Please see amendment to Main Text Results on page 8 line 157.

“Study participants were all people who were alive and permanently registered with 149 included general practices on the study index date, **November 30, 2015**, with least two year’s GP registration prior to this.”

4.      Please mention the funders in the methods.

**Response 45.** Please see **Response 24**.

5.      Please define "IMD" in Table 1.

**Response 46.** Please see addition to Table 1 footnote on page 23.

“****Index of multiple deprivation (IMD)**”

Reviewer #3: Dear editor,
Thank you for allowing me to read such an interesting manuscript.
I believe that the manuscript was well written and that the research was presented in a way that was easy to follow and understand. I think the statistics were adequately explained and their meaning was easy to understand.
I have only one question for the authors: what were the criteria for choosing some condition lists like the ones from Diederichs, Fortin, Ho, Barnett, Salive, but not others that are well know such as O'Halloran and N'Goran (particularly in primary care settings)?
- O'Halloran J, Miller GC, Britt H. Defining chronic conditions for primary care with ICPC-2. Fam Pract. 2004 Aug;21(4):381-6. doi: 10.1093/fampra/cmh407. PMID: 15249526.
- N'Goran AA, Blaser J, Deruaz-Luyet A, Senn N, Frey P, Haller DM, Tandjung R, Zeller A, Burnand B, Herzig L. From chronic conditions to relevance in multimorbidity: a four-step study in family medicine. Fam Pract. 2016 Aug;33(4):439-44. doi: 10.1093/fampra/cmw030. Epub 2016 May 6. PMID: 27154549. – list of conditions in pasted section below

Best regards.

**Response 47.** Thank you for suggesting these additional condition lists and please see addition of the N’Goran condition list. We have not added the O’Holloran condition list because it contains conditions no longer considered chronic (for example “neurological infection” and ‘pelvic inflammatory disease’) and other chronic conditions that did not align to the conditions used in more recent lists (for example ‘weakness/tiredness general’ and ‘neck syndrome’) and therefore resulted in large proportion of missing conditions. We can add the conditions within this list that align to the existing conditions if you think appropriate. The O’Holloran condition list is no longer available online and was obtained from the corresponding author, and we can provide this if needed.

Please see replacement of “**eight** condition lists” with “**nine** condition lists” throughout the manuscript.

Please see addition to Main Text Method section on page 9 line 175.

“Condition-lists were either specifically recommended for measuring multimorbidity (referred to hereinafter as Diederichs,[6] Fortin,[9] Ho always,[10] Ho always + usually[10]**,** **and N’Goran[15])**, used in previous highly-cited large-scale studies of multimorbidity prevalence (Barnett[16] and Salive[17]), or included in widely applied measures of ‘comorbidity’ (Charlson[18] and Elixhauser[19]).

Please see addition to Figure 1, Table 2, Figure 5, Supplementary Table 2.
